# Persisting higher prevalence of hepatitis A virus RNA in blood donors, France, 2018

**DOI:** 10.2807/1560-7917.ES.2019.24.47.1900695

**Published:** 2019-11-21

**Authors:** Pierre Gallian, Valérie Barlet, Lina Mouna, Sylvie Gross, Pascal Morel, Sophie Le Cam, Céline Ricard, Claude Maugard, Elodie Pouchol, Benoit Flan, Catherine Visse, Rachid Djoudi, Julie Figoni, Henriette De Valk, Pierre Tiberghien, Anne-Marie Roque-Afonso

**Affiliations:** 1 Etablissement Français du Sang Provence Alpes Côte d’Azur et Corse, Marseille, France; 2 Unité des Virus Emergents (UVE: Aix-Marseille Univ – IRD 190 – Inserm 1207 – IHU Méditerranée Infection), Marseille, France; 3 Etablissement Français du Sang Auvergne Rhône Alpes, Beynost, France; 4 AP-HP, Hôpital Paul Brousse, Virologie, CNR des Virus des hépatites à transmission entérique, INSERM U1993, Villejuif, France; 5 Etablissement Français du Sang, Saint Denis-La Plaine Stade de France, France; 6 Etablissement Français du Sang Centre Pays de Loire, Nantes, France; 7 Etablissement Français du Sang Haut de France, Lille, France; 8 Etablissement Français du Sang Occitanie, Toulouse, France; 9 LFB Biomédicaments, Courtaboeuf, France; 10 Santé publique France, French national public health agency, Saint-Maurice, France; 11 Université de Franche-Comté, Inserm, Etablissement Français du Sang, UMR 1098, Besançon, France

**Keywords:** France, food-borne infections, viral infections, hepatitis A virus, surveillance

**To the editor:** In the article *Improving preparedness to respond to cross-border hepatitis A outbreaks in the European Union/European Economic Area: towards comparable sequencing of hepatitis A virus* published in *Eurosurveillance* on 11 June 2019, Enkirch et al. emphasised the importance of Europe-wide collaboration to accelerate public health responses for the control of hepatitis A virus (HAV) outbreaks [1]. These collaborations have shown to be effective in the 2016–2017 outbreak mostly affecting men who have sex with men (MSM). In the context of this outbreak, we previously reported increased frequency of HAV-RNA-positive blood donors in 2017 compared with 2015 and 2016 [2]. We hereby update this information and report a persistently higher number of HAV-infected blood donors throughout 2018. In April–June 2017, we observed a peak in the number of HAV-positive blood donors, which was a few weeks earlier than the peak observed in the general population in July. Twelve of the 13 HAV-RNA-positive donors were infected with one of the three genotype IA MSM-associated outbreak viral strains. From the last quarter of 2017, a marked decrease in the number of cases both in the general population and in the blood donors allowed to consider the outbreak as under control in France.

In 2018, however, we unexpectedly detected 13 further HAV-RNA-positive blood donors with one to two cases per month, except in March and November, while the number of notified hepatitis A cases concomitantly decreased by ca 50% in France (1,524 in 2018 vs 3,351 in 2017) and the male /female ratio of infected blood donors decreased from 5.5 in 2017 to 3.3 in 2018, as observed in the general population in France, and elsewhere in Europe [3]. For nine of the 13 positive donations in 2018, viral sequences were obtained and assigned to genotype IA in six cases, including four with MSM-associated outbreak viral strains (vs 12/13 in 2017), and to genotype IB in three cases. Travel to an endemic area during the incubation period was more frequently reported than in 2017 (4 vs 1), including three affected individuals mentioning travel to Morocco and one to Egypt. Three other ones reported seafood exposure; no specific risk factor was identified in the remaining five cases. The persisting higher frequency of HAV-RNA positive blood donors in 2018 may be related to a continued spread of the 2016–2017 outbreak strains in the general population, combined with an increased number of imported cases in travellers returning from areas with ongoing outbreaks such as Morocco (3/12 cases) during the first semester of 2018 [4]. The discrepancy between the higher frequency of HAV-RNA detection in blood donors and the drop in the number of notified HAV cases overall may reflect a return to ‘usual’ levels of under-reporting of cases following the increased hepatitis A awareness during the 2017 outbreak. It is of note that no new cases of HAV-RNA-positive blood donors have been identified for 6 consecutive months from December 2018 to May 2019, suggesting a return to the HAV-RNA prevalence of two and three cases, respectively observed in 2015 and 2016, before the outbreak.

Overall, the risk of collecting blood from an asymptomatic HAV-RNA-positive donor remained higher in 2018 (4.5 HAV-RNA positive donations / 10^6^ tested donations), more than 15 months after the July 2017 peak observed in the general population. This risk resulted in the occurrence in 2018 of two transfusion-transmitted HAV infections involving pathogen-reduced (Intercept, Cerus, Concord, CA, USA) pooled whole blood platelet concentrates. Our data highly suggest that the risk assessment for HAV transmission by substances of human origin should not rely only on general population surveillance data and emphasise the benefit of blood donors’ surveillance for public health purposes. Availability of similar surveys in other European countries would contribute to improving preparedness for hepatitis A outbreaks in Europe and support their control. 
